# Pirfenidone Has Anti-fibrotic Effects in a Tissue-Engineered Model of Human Cardiac Fibrosis

**DOI:** 10.3389/fcvm.2022.854314

**Published:** 2022-03-11

**Authors:** Thomas C. L. Bracco Gartner, Sandra Crnko, Laurynas Leiteris, Iris van Adrichem, Linda W. van Laake, Carlijn V. C. Bouten, Marie José Goumans, Willem J. L. Suyker, Joost P. G. Sluijter, Jesper Hjortnaes

**Affiliations:** ^1^Department of Cardiothoracic Surgery, University Medical Center Utrecht, Utrecht, Netherlands; ^2^Regenerative Medicine Center Utrecht, Circulatory Health Laboratory, Utrecht, Netherlands; ^3^Experimental Cardiology Laboratory, Department of Cardiology, University Medical Center Utrecht, Utrecht, Netherlands; ^4^Department of Biomedical Technology, Eindhoven University of Technology, Eindhoven, Netherlands; ^5^Institute for Complex Molecular Systems (ICMS), Eindhoven University of Technology, Eindhoven, Netherlands; ^6^Department of Cell and Chemical Biology, Leiden University Medical Center, Leiden, Netherlands; ^7^Utrecht University, Utrecht, Netherlands

**Keywords:** cardiac fibrosis, tissue-engineering, disease modeling, pirfenidone, targeted proteomics, 3D cell culture

## Abstract

A fundamental process in the development and progression of heart failure is fibrotic remodeling, characterized by excessive deposition of extracellular matrix proteins in response to injury. Currently, therapies that effectively target and reverse cardiac fibrosis are lacking, warranting novel therapeutic strategies and reliable methods to study their effect. Using a gelatin methacryloyl hydrogel, human induced pluripotent stem cell-derived cardiomyocytes (hiPSC-CM) and human fetal cardiac fibroblasts (hfCF), we developed a multi-cellular mechanically tunable 3D *in vitro* model of human cardiac fibrosis. This model was used to evaluate the effects of a promising anti-fibrotic drug—pirfenidone—and yields proof-of-concept of the drug testing potential of this platform. Our study demonstrates that pirfenidone has anti-fibrotic effects but does not reverse all TGF-β1 induced pro-fibrotic changes, which provides new insights into its mechanism of action.

## Introduction

On a tissue level, heart failure is characterized by cardiomyocyte hypertrophy and apoptosis, and cardiac fibrosis (1). The main effector cell driving cardiac fibrosis is the cardiac fibroblast, which remodels the cardiac extracellular matrix (ECM) upon physiological environmental stimuli. Although cardiac fibroblasts play a pivotal role in the initially beneficial wound healing response by depositing collagens to protect the structural integrity of the cardiac ECM (2), perpetual activation of cardiac fibroblasts during prolonged pathological stress exposure leads to ongoing deposition and accumulation of fibrous ECM material, which eventually causes cardiac dysfunction, most notably diastolic dysfunction, and increased susceptibility for lethal cardiac dysrhythmias (3). Current treatments for heart failure aim at reducing signs and symptoms, but do not target cardiac fibrosis directly (4).

Although many factors are known to be involved, cardiac fibrosis is primarily regulated by transforming growth factor beta (TGF-β). This growth factor activates cardiac fibroblasts into secretory myofibroblasts by inducing formation of α-smooth muscle actin (α-SMA) containing stress-fibers and promoting expression of ECM genes, most notably increasing collagen and periostin levels (5). Although TGF-β is a key factor, treating cardiac fibrosis by targeting TGF-β directly has so far not yielded satisfactory results (4, 6).

The current challenge of targeting cardiac fibrosis directly reveals both our incomplete understanding of this complex pathophysiological process and the need to improve the development of novel cardiovascular drugs (4, 7, 8). An important issue in cardiovascular drug development is the large share of compounds that seem promising in preclinical animal research but fail to show efficacy in human clinical trials (9). To address this challenge, three-dimensional (3D) *in vitro* models of the human heart have been created using tissue-engineering techniques (10). Earlier work by our group established the feasibility of mimicking cardiac fibrosis *in vitro* and has investigated the anti-fibrotic effect of cardiac progenitor cells in 3D tissue-engineered constructs containing human cardiac fibroblasts (11, 12).

The use of 3D tissue-engineered constructs with a physiological ECM stiffness is essential for reliably studying cardiac fibrosis *in vitro*, as cardiac cell behavior is known to be influenced through mechanosensitive pathways (13). Cardiomyocytes have been shown to contract best on hydrogels with an elastic modulus in the physiological range and induced pluripotent stem cell derived cardiomyocytes (iPS-CM) show enhanced maturation and contraction patterns when cultured in a 3D environment which recapitulates the human heart (14, 15). Furthermore, cardiac fibroblasts are known to spontaneously transdifferentiate into their active pro-fibrotic form when exposed to a high substrate stiffness, but to stay quiescent in a 3D environment (16). As such, using a 3D cell culture system with mechanical properties that mimic the human heart seems an important next step to study cardiac fibrosis and develop new anti-fibrotic treatments.

A promising approach with anti-fibrotic effects, clinically used to treat idiopathic pulmonary fibrosis, is pirfenidone (17). Although several preclinical studies have shown its anti-proliferative effect on fibroblasts and decreased deposition of collagens in numerous organs, the precise molecular targets and mechanism of action of pirfenidone are unknown (18–21). The extent of cardiac fibrosis was reduced in rodent models of myocardial infarction (22) and pressure overload (23–25), but to date, the effects of pirfenidone on human cardiac fibrosis are largely unknown.

In this study, we report the optimization of our previously developed 3D tissue-engineered *in vitro* model of human cardiac fibrosis by using a co-culture of induced pluripotent stem cell-derived cardiomyocytes and primary cardiac fibroblasts in a mechanically tunable hydrogel. We demonstrate the feasibility of using this human cardiac fibrosis model as a drug-testing platform, by evaluating the effects of a new anti-fibrotic drug—pirfenidone—on the transcriptomic and proteomic level and providing new insights into its mechanism of action.

## Materials and Methods

### Gelatin Methacryloyl Fabrication

Gelatin methacryloyl (GelMA) was prepared as reported previously (26). Briefly, gelatin type A from porcine skin (Sigma-Aldrich) was dissolved in PBS (10% w/v) under continuous stirring for 20 min at 60°C. Gelatin molecules were modified by adding methacryloyl side groups through dropwise addition of 8% (v/v) methacrylic anhydride at 50°C for 3 h. The reaction was stopped by adding PBS. The GelMA solution was then dialyzed in 12–14 kDa dialysis tubing for a week. Afterwards, the GelMA solution was put through a 22 μm filter, lyophylized for a week and stored at −80°C until further use. H-NMR was used to confirm the degree of methacrylation.

### Hydrogel Characterization

The elastic modulus of GelMA hydrogels was assessed through a micro-indentation test, applying unconfined compression at a constant rate (0.1 mm/s) up to a strain of 70% at RT. The elastic modulus was then calculated from the linear region of the stress-strain curve.

### Cell Sources

Healthy fetal cardiac tissue was transferred to the laboratory for experimental use after planned abortions. Parental consent was given for all fetal material involved and the protocol has been approved by the Medical Ethical Committees of the University Medical Center Utrecht and Leiden University Medical Center, as previously described (12, 27). All procedures are in accordance with the declaration of Helsinki (on ethical principles for medical research involving human subjects) and the declaration of Taipei (on ethical considerations regarding health databases and biobanks) (28, 29).

### Human Fetal Cardiac Fibroblast (HfCF) Isolation and Culture

Single cell suspension of heart tissue was plated overnight on uncoated standard tissue culture plastic to allow fibroblasts to adhere. Human fetal cardiac fibroblasts (hfCF) were cultured using fibroblast medium, consisting of Dulbecco's Modified Eagle Medium (DMEM, Gibco 41965), supplemented with 10% fetal bovine serum (FBS, Biowest S1810) and 1% penicillin/streptomycin (Gibco 15140122). HfCF were expanded in culture, passaged at a confluency of 90% and harvested for experimental use at passage 4-8. Cells were maintained at 5% CO_2_, 20% O_2_, 37°C, in a humidified atmosphere.

### Human Induced Pluripotent Stem Cell-Derived Cardiomyocyte (iPS-CM) Differentiation and Culture

All iPS cell lines were provided by the European Bank for Induced Pluripotent Stem Cells. iPS cells were cultured on 10 μg/cm^2^ Matrigel-coated plates (Corning 356231) and maintained in E8 medium (Gibco A1517001). When iPS cells reached confluency (~70–80%) they were either split using 0.5 mM EDTA (Invitrogen 15575020) or differentiated toward cardiomyocytes. Differentiation was started using CHIR-99021 (Selleck Chemicals S2924) in heparin medium (DMEM/F12 (Gibco 31331) containing 1:100 chemically defined lipid concentrate (Gibco 11905031), 213 μg/ml L-ascorbic acid (Sigma-Aldrich A8960), 1.5 IU/ml heparin (LEO 9005496) and 1% penicillin/streptomycin). After 48 h, the medium was replaced by heparin medium containing 2 μM WNT-C59 (R&D Systems 5148). Another 48 h later, the medium was replaced by heparin medium. From day 7 until day 10 the medium was replaced by insulin medium [DMEM/F12 containing 1:100 chemically defined lipid concentrate, 213 μg/ml L-ascorbic acid, human recombinant insulin (Sigma-Aldrich I9278) and 1% penicillin/streptomycin]. Starting on day 10, the cells were purified using purification medium [RPMI 1640 L-glutamine without glucose (Gibco 11879), 1:100 chemically defined lipid concentrate, 213 μg/ml L-ascorbic acid, 21 μg/ml human recombinant insulin, 3.5 μM sodium-DL-lactate (Santa Cruz Biotechnology 301818) and 1% penicillin/streptomycin]. On day 15 the cells were replated using Tryple Select 10x (Thermo Fisher Scientific A1217702), after which they were maintained in RPMI 1640 L-glutamine (Gibco 21875) containing 0.5x B27 (Thermo Fisher Scientific 17504001). Only batches with an iPS-CM purity above 95% were used for experiments.

### Tissue-Engineering Cardiac Tissue Constructs

**Figure 1:**
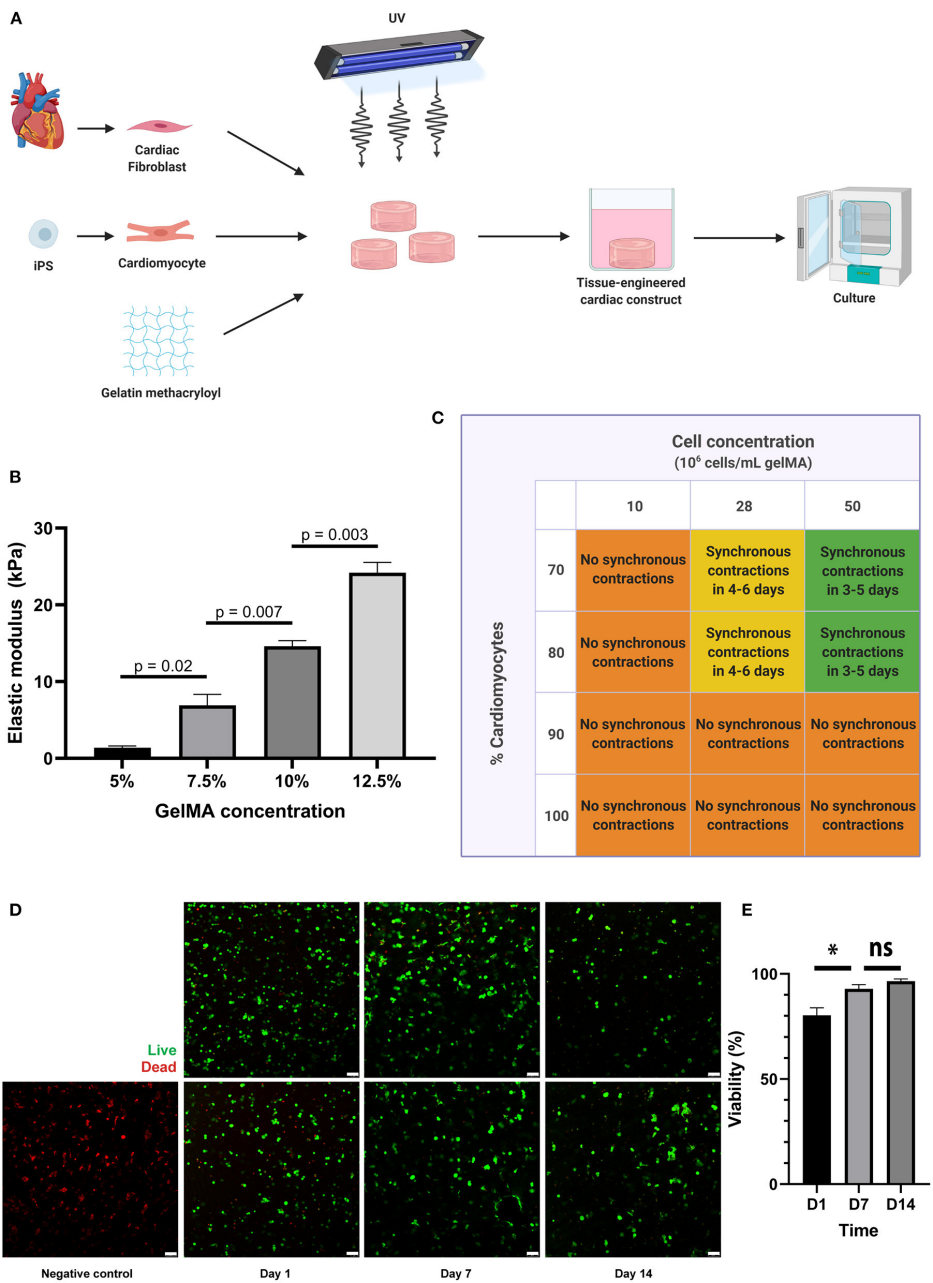
A 3D co-culture of cardiac fibroblasts and iPS-derived cardiomyocytes in a GelMA hydrogel results in a functional cardiac tissue construct. **(A)** Schematic overview of the tissue engineering process. Primary cardiac fibroblasts and iPS-CM were suspended in a GelMA polymer solution and subsequently cross-linked by exposure to UV-light (wavelength 365 nm). The resulting cell-laden hydrogels were washed in PBS to remove uncrosslinked polymers and were then placed in culture medium in non-adhesive well plates for suspension culture. **(B)** Mechanical characterization of the hydrogels, showing the elastic modulus (in kPa) for different GelMA concentrations as measured by microindentation testing (*n* = 3 per condition). Statistical analysis was performed using a one-way ANOVA with Sidak's multiple comparison test. Data are represented as mean ± SEM. **(C)** Schematic overview of the outcomes of combined cell density and cell ratio optimization experiments. Synchronous contractions throughout the cardiac tissue constructs were only observed when a cell concentration of 28 or 50 million cells per milliliter was used, with a cardiomyocyte percentage of 70 or 80. In other conditions, individual cells were contracting, but synchronous contractions throughout the cardiac tissue construct would not develop. **(D,E)** Cells remain viable in 7.5% GelMA until 14 days in culture, as demonstrated by immunofluorescence microscopy after live-dead staining on day 1, day 7, and day 14 after cardiac tissue construct fabrication (*n* = 3). Scale bars, 75 μm. *p < 0.05, ns, non-significant.

GelMA was used as a scaffold material for 3D culture of hfCF and iPS-CM (Figure 1A). First, photo-initiator (PI, Irgacure 2959) was dissolved in PBS in a concentration of 0.1% (w/v). GelMA was added to the PI-solution in concentrations of 7,5% and 10% (w/v). Simultaneously, hfCF and iPS-CM had been harvested and the cells were resuspended in the GelMA polymer solution. A 30 μL drop of cell-laden GelMA polymer solution was placed between two 450 μm high spacers and covered by a glass slide. Crosslinking of the polymer solution was then induced by UV-light (wavelength 365 nm, 5.6 W/cm^2^ for 50 s), resulting in cardiac tissue constructs (CTC). CTC were washed in PBS once, put in non-adhesive well plates for suspension culture and maintained at 5% CO_2_, 20% O_2_, 37°C, in a humidified atmosphere. CTC were maintained in DMEM containing 10% KnockOut serum replacement (Gibco 10828028) and 1% penicillin/streptomycin.

### Experimental Conditions

CTC were engineered at day 0 and allowed to self-organize during a week until synchronous beating was observed. On day 7, experimental conditions were started (TGF-β1 and/or pirfenidone vs. control) and the constructs were harvested on day 14 for subsequent analysis. Transforming growth factor beta 1 (TGF-β1, Peprotech 100-21C) was used in a concentration of 2 ng/mL to induce a fibrotic response, as reported in earlier work (11, 12). Pirfenidone (Cayman Chemical 13986) was used to treat fibrosis in a concentration of 1 mg/mL, in accordance with work of other groups (18, 21, 30). Medium was renewed every 2 days.

### Viability Assay

Cell viability in CTC was assessed using the Live/Dead Viability kit for mammalian cells (Life Technologies). CTC were washed twice with PBS and subsequently incubated for 30 min at room temperature in a 2 μM calcein-AM and 4 μM ethidium homodimer-1 solution. After incubation, CTC were washed with PBS and immediately imaged using a Leica SP8X confocal microscope. Z-stacks (step size 10 μm) were made at 3 random sites per CTC and quantified using ImageJ software.

### RNA Isolation and CDNA Synthesis

CTC were frozen at −80°C in 1 mL TriPure isolation reagent (Roche). After thawing, the samples were homogenized using ceramic beads (1.4 mm zirconium oxide beads, Precellys) and a beadbeater. The lysate was transferred to a new Eppendorf tube, which was centrifuged at 12,000 g to remove debris. Chloroform was added to the supernatant, vortexed and subsequently centrifuged at 12,000 g. The aqueous layer was loaded on an RNA isolation column (NucleoSpin RNA columns, Macherey-Nagel) with 70% ethanol. RNA was isolated using manufacturer's instructions, which included DNase treatment (RNase-free DNase set, Qiagen). Isolated RNA was quantified using a DS-11 spectrophotometer (DeNovix) and 100 ng was taken to synthesize cDNA (qScript cDNA synthesis kit, QuantaBio).

### Polymerase Chain Reaction (PCR)

Quantitative real-time PCR was performed in a BioRad CFX Connect, using SYBR Green (QuantaBio) and specific primers for genes of interest, including GAPDH, periostin, α-SMA and COL1a1. Primer sequences can be found in Supplementary Table 1. Relative expression of genes was quantified using the 2^−*ddCt*^ method (31).

### Histology and Immunofluorescence Staining

CTC were washed twice in PBS and fixed in 4% paraformaldehyde (Santa Cruz Biotechnology 281692) for 25 min. CTC were partly dehydrated in a 30% (w/v) sucrose solution overnight at 4°C before being embedded in TissueTek OCT Compound (Sakura 4583). Cryosections of 7 μm were made using a cryotome (Thermo fisher Cryostar NX70). The slides were dried for 1 h at room temperature and rehydrated with PBS for 10 min. Samples were permeabilized using 0.1% triton (Sigma-Aldrich X-100) for 10 min, washed thrice for 5 min using 0.5% Tween-80 (Millipore 817061) and blocked using 5% BSA (Millipore 10735086001) for 30 min. Slides were washed again and subsequently incubated with primary antibodies (Supplementary Table 2) diluted in 5% BSA for 90 min at room temperature. After washing, secondary antibodies were combined with 1 μg/ml Hoechst 33342 (Invitrogen H1399) and incubated for 60 min at room temperature. Afterwards, slides were washed with PBS thrice for 5 min and sealed with Fluoromount-G (Invitrogen 00495802). Slides were imaged using a Leica SP8X confocal microscope and analyzed using ImageJ software.

### Calcium Transient Imaging

A 2.5 μM solution of Cal520 (Abcam) in 90% Fluorbright DMEM (Gibco A1896701) supplemented with 10% F127 was made and warmed to 37°C. The culture medium of CTC was diluted with the Cal520 solution in a 1:1 ratio to reach a concentration of 1.25 μM and left to incubate for 1 h at 37°C. Calcium transients in CTC were subsequently imaged using a Leica SP8X confocal microscope.

### Protein Isolation and Targeted Proteomics

CTC were washed twice in PBS, after which each hydrogel was incubated at 37°C in TrypLE Select (Gibco 12604) for 30 min. To completely degrade the CTC, Liberase TH (Roche 05401135001) dissolved in HBSS (Gibco 24020) was added and the lysate was incubated for another 30 min at 37°C. Protein content of the lysate was analyzed using the BCA Protein Assay Kit (Thermo Fisher 23225) and targeted proteomics was performed using Cardiovascular Panel 3 of Olink Proteomics (Uppsala, Sweden).

### Statistics

Results were analyzed using Graphpad Prism software (version 7.02, La Jolla, California, USA). Means are reported with the standard error of the mean (SEM), unless indicated otherwise. Paired two-tailed *t*-tests were used to compare the means of two groups and a two-way ANOVA for repeated measures with a Tukey's multiple comparisons *post-hoc* test was used to compare the means of multiple groups. A *p*-value < 0.05 was considered statistically significant. Figures were created using Adobe Illustrator and BioRender.com.

## Results

### A 3D Co-culture of Cardiac Fibroblasts and iPS-Derived Cardiomyocytes in a GelMA Hydrogel Results in a Functional Cardiac Tissue Construct

In this study, we aimed to use an *in vitro* model of human cardiac fibrosis using GelMA, hfCF and iPS-CM (Figure 1A). First, the mechanical characteristics of 5, 7.5, 10, and 12.5% GelMA were tested by micro-indentation and revealed elastic moduli of 1.4 to 24.2 kPa (Figure 1B), which is in line with previous reports (32). The elastic modulus of 7.5% GelMA was 6.9 ± 1.4 kPa and mimics the stiffness of the healthy heart (33). Therefore, in order to reliably represent the *in vivo* elastic modulus, and based on the excellent cell viability within the construct, this GelMA concentration was selected for all subsequent experiments involving cardiac cells.

Furthermore, to enable essential cell-cell contact and coupling throughout the cardiac tissue constructs (CTC), we varied the cell density and the ratio between iPS-CM and hfCF and monitored synchronous beating. Cell densities of 10 million cells per milliliter, 28 million cells per milliliter and 50 million cells per milliliter were compared. Simultaneously, different ratios between iPS-CM and hfCF were used, in which constructs contained 70, 80, 90, or 100% iPS-CM and 30, 20, 10, and 0% hfCF, respectively (Figure 1C). CTC with 100 and 90% iPS-CM did not demonstrate synchronous contractions (Supplementary Video 1), irrespective of cell density used. For CTC containing 10 million cells per milliliter, no synchronized contractions were noted either (Supplementary Video 2), irrespective of cell ratios used. However, the CTC consisting of 28 and 50 million cells per milliliter with 70 or 80% iPS-CM did show synchronized beating throughout the entire construct (Supplementary Videos 3, 4).

As commonly seen for tissue engineered constructs, cells need additional time to become fully functional after fabrication (10, 34, 35). In this study, we refer to this period as the maturation phase. The iPS-CM started contracting individually within 2 to 5 days after embedding and would progress to contracting synchronously throughout the CTC within 1 or 2 additional days. However, only when cell concentrations of 28 or 50 million cells per milliliter were used with 70 or 80% iPS-CM. CTC started contracting synchronously earlier when a cell concentration of 50 million cells per milliliter was used and were consistently doing so before day 6. The functional coupling of cells throughout the construct and the resulting electromechanical coupling could be visualized by imaging calcium transients with a fluorescent calcium dye (Supplementary Video 5). Furthermore, CTC showed excellent cell viability until 14 days after fabrication (Figures 1D,E), with an average cell viability of 80% on day 1, 93% on day 7, and 97% on day 14. All subsequent experiments were therefore performed in CTC containing 70% iPS-CM with a cell density of 50 million cells per milliliter.

### TGF-β1 Induces a Fibrotic Response in Cardiac Tissue Constructs

**Figure 2:**
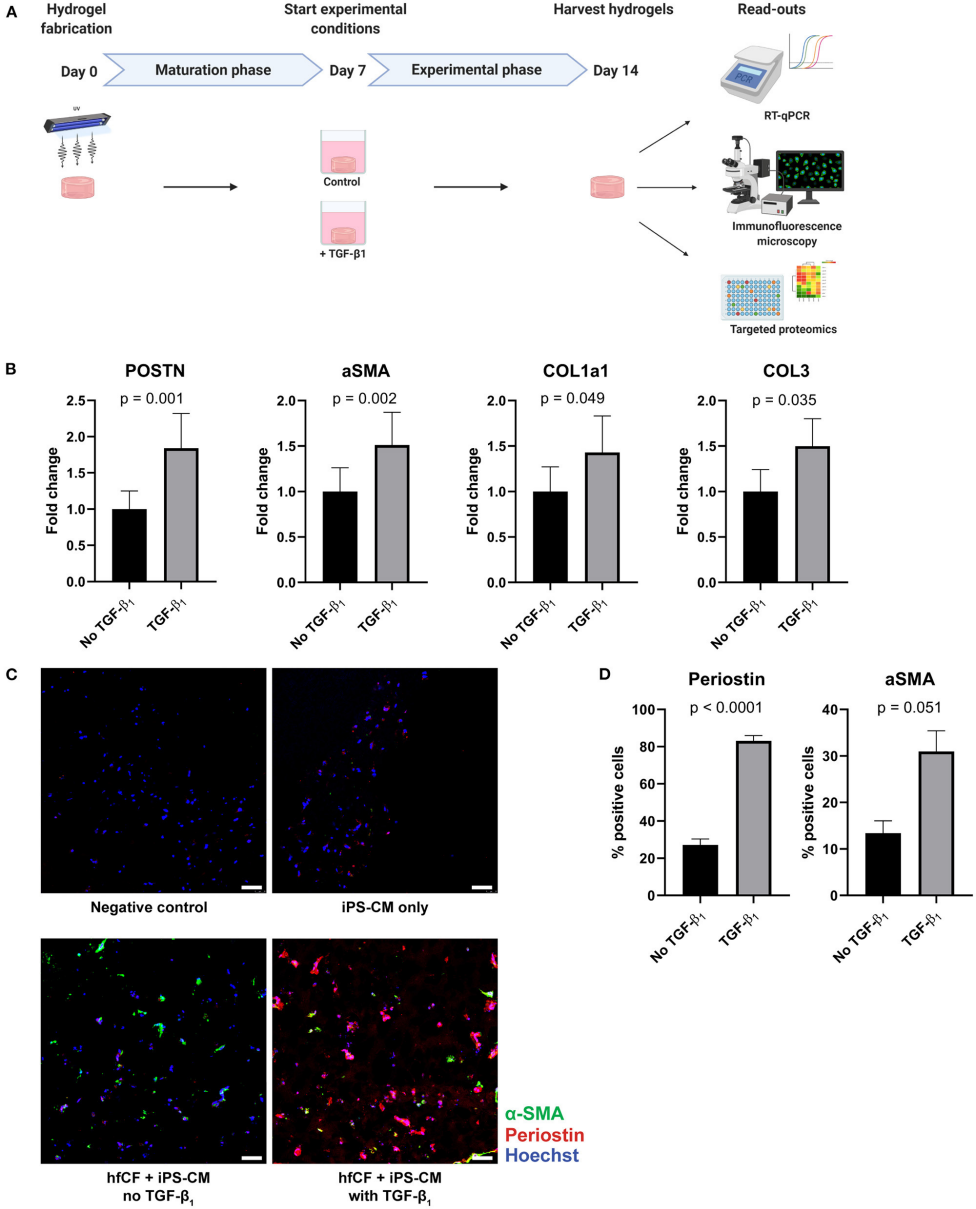
TGF-β1 induces a fibrotic response in cardiac tissue constructs. **(A)** Timeline of the experiment. Cardiac tissue constructs were fabricated at day 0 and cultured until day 7. On day 7, experimental conditions were started. Two experimental conditions were created: TGF-β1 was added in the first group in a concentration of 2 ng/mL, the other group served as controls. On day 14, the constructs were harvested for analysis, consisting of RT-qPCR, immunofluorescence staining and targeted proteomics. **(B)** TGF-β1 stimulation results in an increased expression of fibrotic genes periostin (POSTN), alpha-smooth muscle actin (aSMA), collagen type 1 (COL1a1), and collagen type 3 (COL3), as demonstrated by RT-qPCR (*n* = 13). Data are represented as mean relative expression (compared to GAPDH) ± SEM. Statistical analysis was performed using paired two-tailed Student's *t*-test. **(C,D)** Immunofluorescence staining shows an increase in α-SMA (green) and periostin (red) expression in tissue-engineered cardiac constructs stimulated with TGF-β1 (*n* = 4). Scale bars, 50 μm. Statistical analysis was performed using paired two-tailed Student's *t*-test.

To examine the potential of CTC to serve as an *in vitro* model for human cardiac fibrosis, TGF-β1 was added to the culture medium from day 7 onwards in a concentration of 2 ng/mL (Figure 2A). This resulted in an increase in expression of well-established fibrotic markers on day 14; α-SMA (*p* = 0.002), periostin (POSTN, *p* = 0.001), collagen type 1 (COL1a1, *p* = 0.049), and collagen type 3 (COL3, *p* = 0.035) as measured by RT-qPCR (Figure 2B). Immunofluorescence imaging confirmed the increase in periostin (27% vs. 83%, *p* < 0.0001) and α-SMA (13% vs. 31%, *p* = 0.051) expression after stimulation with TGF-β1 (Figures 2C,D).

**Figure 3:**
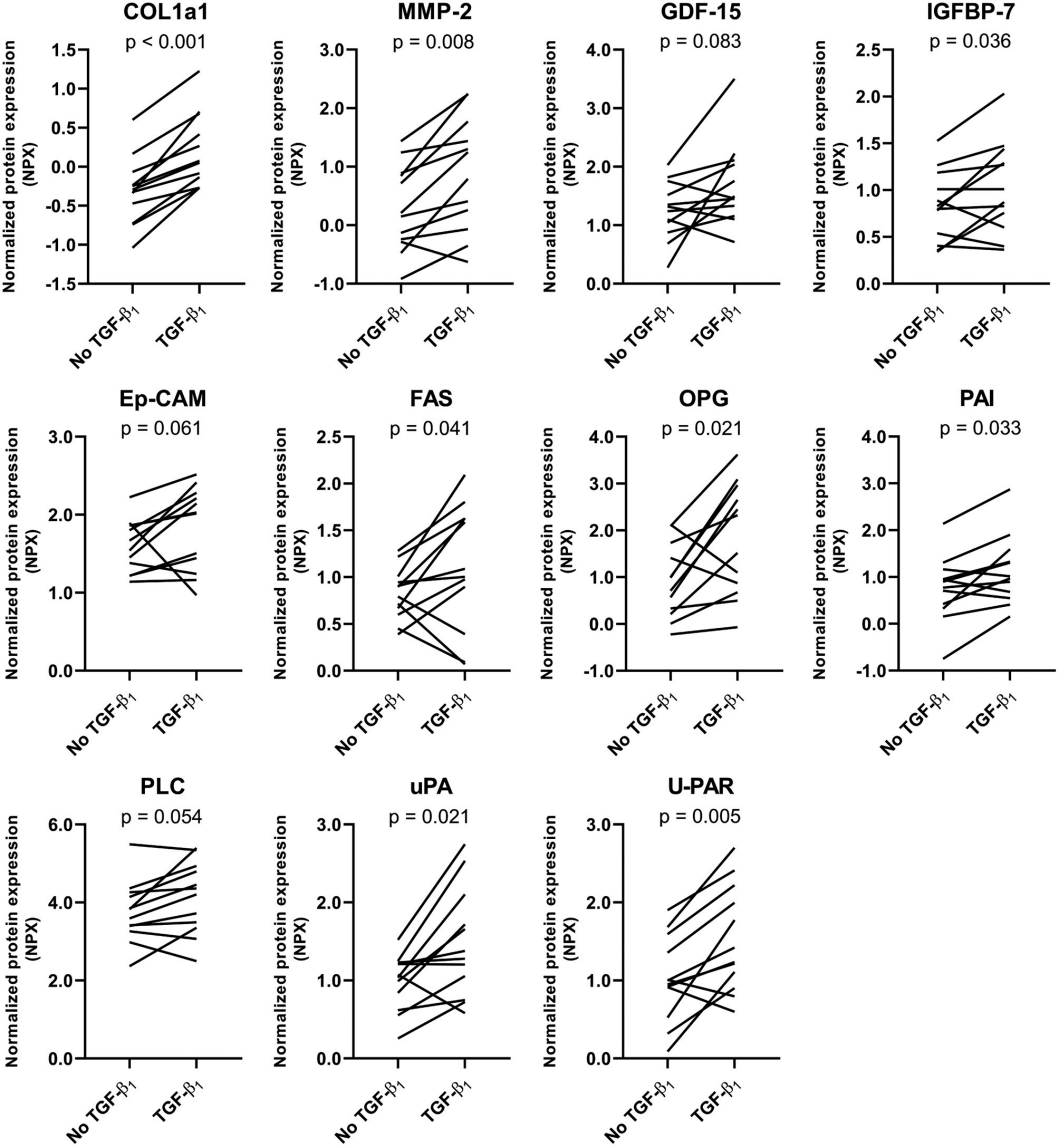
Targeted proteomics confirms the TGF-β1-induced fibrotic response in tissue-engineered cardiac constructs. TGF-β1 stimulation results in an increased expression of several fibrosis-related proteins. Data are represented as mean normalized protein expression (compared to internal control) per experiment (*n* = 12). Statistical analysis was performed using paired two-tailed Student's *t*-test.

To further investigate the fibrotic effects of TGF-β1 on CTC, we performed targeted proteomics in which a preset panel of 92 cardiovascular disease-related proteins was measured in CTC lysates. Twenty seven of these proteins were detected in our CTC upon TGF- β1 stimulation. TGF-β1 caused an increase in expression of key fibrotic proteins COL1a1 (*p* = 0.0003, Figure 3), matrix metalloproteinase-2 (MMP2, *p* = 0.008) and osteoprotegerin (OPG, *p* = 0.021), and in heart failure-associated proteins insulin-like growth factor binding protein-7 (IGFBP7, *p* = 0.036) and growth differentiation factor-15 (GDF-15, *p* = 0.08). Also, upon TGF-β1 stimulation, cardiac hypertrophy associated protein phospholipase C (PLC) and epithelial cell adhesion molecule (Ep-CAM), involved in epithelial-mesenchymal transition (EMT), showed a trend toward increased expression (*p* = 0.054 and *p* = 0.061, respectively). Furthermore, expression of tumor necrosis factor superfamily member 6 (FAS) was increased (*p* = 0.041), as were three plasminogen related proteins, including plasminogen activator inhibitor (PAI, *p* = 0.033), urokinase-type plasminogen activator (uPA, *p* = 0.021) and urokinase receptor (U-PAR, *p* = 0.005). Overall, TGF-β1 induces distinct pro-fibrotic and heart failure related changes, establishing CTC as a relevant cardiac fibrosis model.

### Pirfenidone Has Distinct Anti-fibrotic Effects in Cardiac Tissue Constructs

**Figure 4:**
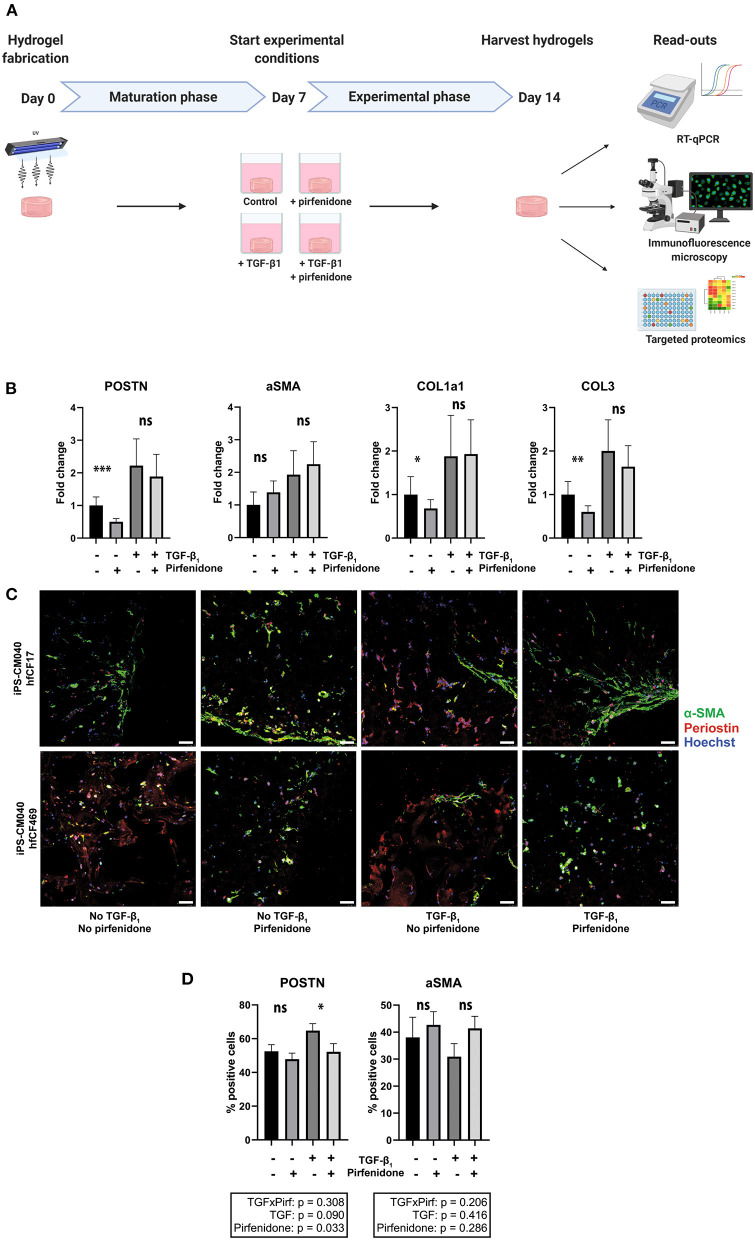
Pirfenidone has distinct anti-fibrotic effects in cardiac tissue constructs. **(A)** Timeline of the experiment. Cardiac tissue constructs were fabricated at day 0 and cultured under regular circumstances until day 7. On day 7, experimental conditions were started. Four experimental conditions were created, in which TGF-β1 (2 ng/mL) and/or pirfenidone (1 mg/mL) were added to the culture medium and a group without TGF-β1 and pirfenidone served as controls. On day 14, the constructs were harvested for analysis, consisting of RT-qPCR, immunofluorescence staining and targeted proteomics. **(B)** Pirfenidone causes a decrease in periostin (POSTN), collagen type 1 (COL1a1) and collagen type 3 (COL3) expression in control conditions, but does not affect alpha-smooth muscle actin (aSMA) expression, as assessed with RT-qPCR (*n* = 13). Data are represented as mean relative expression (compared to GAPDH) ± SEM. Statistical analysis was performed using repeated measures two-way ANOVA. The interaction effect and the main effects are reported in the statistical box underneath the graph, the simple main effects are reported in the graph itself. **(C,D)** Immunofluorescence staining shows a decrease in periostin (red) expression after pirfenidone treatment of cardiac tissue constructs, whereas α-SMA (green) was unaffected (*n* = 11). Each row shows a separate experiment with different cell lines included. Scale bars, 50 μm. Statistical analysis was performed using repeated measures two-way ANOVA. The interaction effect and the main effects are reported in the statistical box underneath the graph. *p < 0.05, **p < 0.01, ***p < 0.001, ns, non-significant.

The anti-fibrotic drug pirfenidone was added to conditions with and without TGF-β1 and these were compared to respective controls (Figure 4A). Pirfenidone resulted only in a reduction of periostin, COL1a1 and COL3 mRNA expression (*p* < 0.001, *p* = 0.011, *p* = 0.002) in the absence of exogenous TGF-β1 (Figure 4B). In the presence of exogenous TGF-β1, pirfenidone did not have an anti-fibrotic effect on the explored mRNAs (*p* = 0.55, *p* = 0.99, *p* = 0.30). Activation status of fibroblasts, explored via α-SMA expression or immunofluorescence imaging was not significantly affected by pirfenidone in either condition (*p* = 0.63 with exogenous TGF-β1 and *p* = 0.10 without exogenous TGF-β1). However, periostin expression decreased in our human CTC upon pirfenidone treatment (64% vs. 55%, *p* = 0.03) (Figures 4C,D).

**Figure 5:**
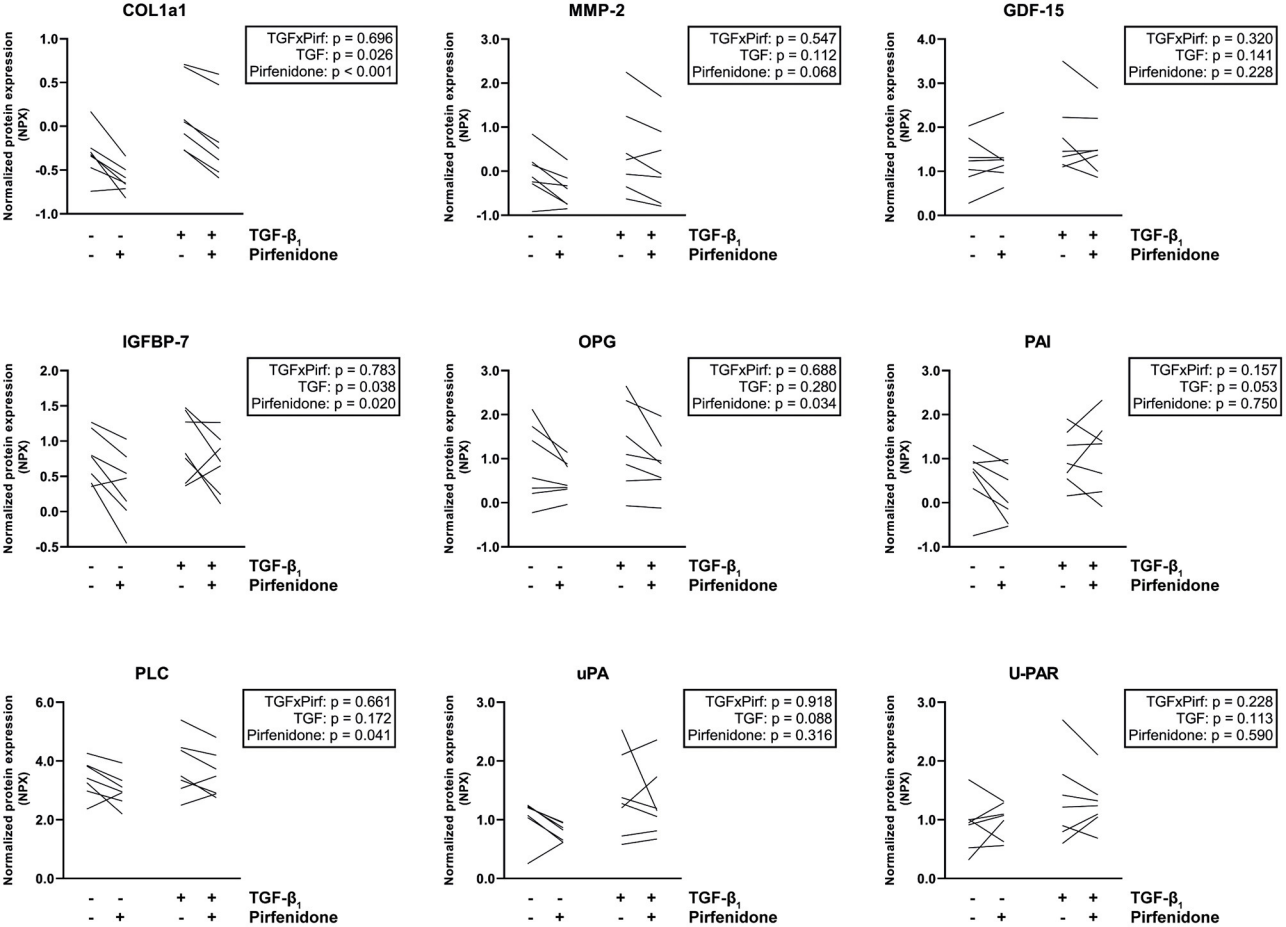
Targeted proteomics reveals that pirfenidone does not undo all TGF-β1-induced pro-fibrotic effects. Pirfenidone causes a decrease in COL1a1 expression in both fibrotic and control conditions. In other fibrosis and heart failure related proteins, different effects can be seen. Data are represented as mean normalized protein expression (compared to internal control) per experiment (*n* = 7). Statistical analysis was performed using repeated measures two-way ANOVA. The interaction effect and the main effects are reported in the statistical box on the right side of the graph.

To confirm these observations and explore which specific effects can be seen upon pirfenidone exposure, we repeated the targeted proteomics approach. Twenty seven of these proteins were detected in CTC lysates. Pirfenidone caused a decrease in collagen type 1, MMP2 and OPG expression in both the presence and the absence of TGF-β1 (*p* < 0.001, *p* = 0.068, *p* = 0.034, Figure 5). Both heart failure associated IGFBP7 expression (*p* = 0.02) and cardiac hypertrophy associated PLC expression (*p* = 0.041) showed a similar decrease. However, pirfenidone did not cause a decrease in expression of heart failure related protein GDF-15, nor did it significantly influence the expression of plasminogen related proteins PAI, uPA and U-PAR which were all upregulated by TGF-β1 stimulation.

Overall, pirfenidone demonstrated distinct anti-fibrotic effects in our CTC, but did not reverse all TGF-β-induced changes.

## Discussion

This study demonstrates that a tissue-engineered 3D *in vitro* model of human cardiac fibrosis can be used as a drug screening platform to investigate anti-fibrotic properties of new cardiovascular drug candidates. Using this human cardiac fibrosis model, we investigated the effects of pirfenidone, an anti-fibrotic drug that showed promising results in pre-clinical animal models of cardiac fibrosis (23, 25, 36) and is already used clinically in idiopathic pulmonary fibrosis (17). This study demonstrated that pirfenidone can have favorable anti-fibrotic effects *in vitro* in human cardiac fibrosis as well but does not undo all the TGF-β1-induced changes in cardiac cell behavior.

Employing a 3D cell-culture system for this study was essential, as these have been proven superior to cell monolayers when mimicking the functions of living tissues (37, 38). In this study, GelMA was utilized as the hydrogel of choice due to its favorable and well-established properties for 3D cell-culture (26, 32). Most importantly, GelMA has a controllable and tunable stiffness; via modification of the methacrylation degree, gel concentration and exposure to UV-light, its mechanical properties can be adjusted to suit the cell type in question (26).

Furthermore, ECM characteristics have been shown to strongly influence cellular functionality and behavior (39). As demonstrated here, hiPSC-CM embedded in GelMA hydrogels were fully functional and seemed to be interconnected, exhibiting synchronous beating throughout the constructs. Two main cardiac cell types were used here, namely iPS-derived cardiomyocytes and cardiac fibroblasts; however, based on this successful co-culture, our system could allow for the integration of other relevant cell types as well, such as endothelial or immune cells. The importance of multicellular constructs is shown in this study, where cardiomyocytes needed to be surrounded by a certain number of cardiac fibroblasts in order to functionally connect and to start beating synchronously. This is in line with other studies in the field, which show that cardiomyocytes, in co-culture with cardiac fibroblasts, have a more mature phenotype, align better with their environment, and start beating synchronously (10, 35, 40).

Upon continuous TGF-β1 stimulation, an evident fibrotic response was observed on both the transcriptomic and proteomic level. Expression of major pro-fibrotic genes α-SMA, POSTN, COL1a1 and COL3 was significantly elevated compared to controls, which is in line with previously published studies (12, 34). While in previous fibrosis research the focus was mainly on RNA or microRNA expression, we focused on the protein level and for the first time utilizing a targeted proteomics approach in which a panel of 92 cardiovascular disease-related proteins was measured in a human *in vitro* model of cardiac fibrosis. TGF-β1 induced the expression of key fibrotic proteins collagen type 1 and MMP2, but also less well-known heart failure related proteins, such as OPG, IGFBP7, PAI, uPA, and U-PAR.

uPA is a serine protease which is associated with tissue remodeling and cell migration. When bound to its cell surface receptor U-PAR, it has extracellular proteolytic activity and can activate MMP's (41). In a recent study, uPA and U-PAR, along with PAI, have been identified as strong predictors of adverse cardiovascular outcomes in chronic heart failure (42).

PAI has been known to be upregulated by TGF-β but has also been proposed to be cardioprotective in rodent models, making its exact role in cardiac fibrosis unclear (43, 44). A recent study clarifies this controversial matter. It identifies PAI as a molecular switch which controls the heart's TGF-β axis through a cardiomyocyte specific feedforward mechanism in which PAI induces TGF-β production and cardiac fibrosis (45). The cardiomyocyte specificity of this mechanism stresses the importance of including cardiomyocytes in cardiac fibrosis *in vitro* models.

Interestingly, some of the upregulated cardiac fibrosis and heart failure related proteins could be treated by administration of pirfenidone. The expression of key fibrotic protein COL1a1 was decreased upon treatment, confirming the status of pirfenidone as a potential cardioprotective anti-fibrotic drug. Another gene directly related to ECM remodeling is MMP2, of which the expression was also reduced by pirfenidone treatment. MMPs play an important role in cardiac remodeling by causing proteolytic degradation of the ECM. The upregulation of MMP2 by TGF-β1 could therefore point to ECM degradation, with its subsequent downregulation due to pirfenidone treatment preventing ECM degradation. However, the precise effect of pirfenidone on ECM turnover *in vivo* is still hard to predict. The overall effect depends on a complex interaction of different components, including the presence and activation of MMPs, the presence of and interaction with their natural inhibitors (TIMPs), the ongoing collagen production and crosslinking, and the complex interplay between MMPs and ECM signaling. Furthermore, the roles of individual MMPs in human cardiac fibrosis are not fully elucidated yet. Nonetheless, the decrease in MMP2 expression by pirfenidone does underline its TGF-β antagonizing effect.

**Figure 6:**
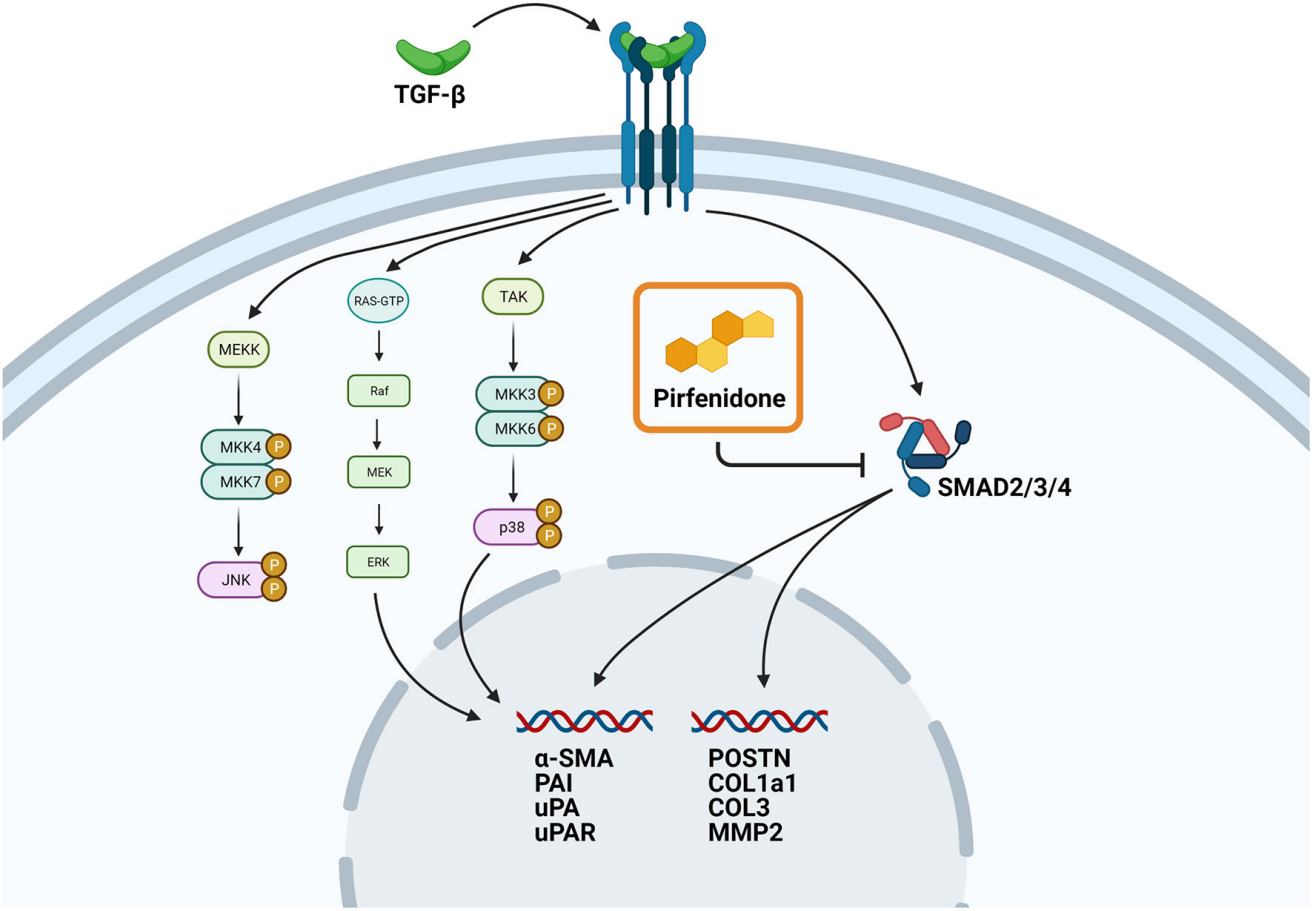
Proposed mechanism of action of pirfenidone. Schematic overview showing the proposed role of pirfenidone in CTC. Upregulation of pro-fibrotic gene expression through the canonical TGF-β pathway is blocked by pirfenidone, but upregulation of pro-fibrotic gene expression through the non-canonical pathway is still possible.

However, not all the TGF-β1 induced changes in protein expression were reversed. Whereas, COL1a1, MMP2, OPG, and IGFBP7 expression were reduced by pirfenidone treatment, the plasminogen related proteins PAI, uPA and U-PAR were not affected. This is in line with a recent study in engineered human heart tissues which showed a reduction in gene expression in several fibrotic genes upon pirfenidone treatment, but not in α-SMA expression (34). A possible explanation for this observation is that pirfenidone interferes in the canonical TGF-β pathway but not in the non-canonical TGF-β pathways (Figure 6).

A convincing body of evidence in preclinical research supports the endeavor of the recently started PIROUETTE trial which studies the anti-fibrotic properties of pirfenidone in HFpEF patients, even though the exact mechanism of action of pirfenidone has not been elucidated yet (46, 47). *In vitro* models of human cardiac fibrosis could play an important role in clarifying the molecular basis of the results found in this clinical study, as it is difficult to study the effects of pirfenidone treatment in the cardiac tissue of the included patients.

The rapid increase of new heart failure cases urges a necessary shift in the current approaches. Although conventional monolayer cell-culture systems and animal studies gave us valuable information about cardiac physiology and the changes occurring during pathological remodeling, these models do not facilitate the translation of effective therapeutics to the clinical arena when it comes to targeting cardiac fibrosis. Advanced human *in vitro* models could bridge this gap by providing us with the necessary tissue complexity without the disadvantages of interspecies differences.

In this study we engineered a 3D *in vitro* model which recapitulates human cardiac fibrosis. By successfully incorporating hiPSC-CM derived from various donors, we opened a window of opportunity toward a more personalized approach to tackling heart failure. With the recent successful creation of hiPSC-cardiac fibroblasts (48, 49), the future 3D fibrosis models will be able to completely mimic patient-specific situations, allowing for tailored drug-testing. Our model can be used for identification of differential fibrosis-related transcriptomic and proteomic profiles in diseased and healthy cells, as well as to screen and test novel anti-fibrotic therapeutics, proof-of-principle of which we provided with pirfenidone. Furthermore, by using this tunable 3D cell-culture system new therapeutic targets could be found, ultimately contributing to the development of interventions that could prevent or reverse fibrotic changes in the failing heart.

## Data Availability Statement

The raw data supporting the conclusions of this article will be made available by the authors, without undue reservation.

## Author Contributions

Experiments were performed by TB, SC, LL, and IA. TB, SC, JS, and JH interpreted data. TB and SC did statistical analysis and wrote the manuscript. LL, CB, MG, WS, JS, and JH edited the manuscript. All authors have read and agreed to the content of the manuscript.

## Funding

We acknowledge the support from Innovation and the Netherlands CardioVascular Research Initiative (CVON): The Dutch Heart Foundation (2017T040), Dutch Federation of University Medical Centers, the Netherlands Organization for Health Research and Development, and the Royal Netherlands Academy of Science. Additionally, the ZonMW Translational Adult Stem Cell grant 1161002016 and Horizon2020 ERC-2016-COG EVICARE (725229).

## Conflict of Interest

The authors declare that the research was conducted in the absence of any commercial or financial relationships that could be construed as a potential conflict of interest.

## Publisher's Note

All claims expressed in this article are solely those of the authors and do not necessarily represent those of their affiliated organizations, or those of the publisher, the editors and the reviewers. Any product that may be evaluated in this article, or claim that may be made by its manufacturer, is not guaranteed or endorsed by the publisher.
