# Supplementary material for: Pirfenidone Has Anti-fibrotic Effects in a Tissue-Engineered Model of Human Cardiac Fibrosis
Source: Front Cardiovasc Med. 2022 Mar 11;9:854314. doi: 10.3389/fcvm.2022.854314 (PMC8963358; doi:10.3389/fcvm.2022.854314)
Supplement: Supplementary file 1 [file Data_Sheet_1.docx]

Supplementary Material

**Supplementary Video 1 |** No synchronized contractions in cardiac tissue constructs when no cardiac fibroblasts are present (cell density of 28 million cells/mL, 100% iPS-CM, 0% hfCF, GelMA 7.5%, imaging at day 7).

**Supplementary Video 2 |** No synchronized contractions in cardiac tissue constructs with a cell density of 10 million cells/mL at day 6 (70% iPS-CM, 30% hfCF, GelMA 7.5%).

**Supplementary Video 3 |** Synchronized contractions in cardiac tissue constructs with a cell density of 28 million cells/mL at day 6 (70% iPS-CM, 30% hfCF, GelMA 7.5%).

**Supplementary Video 4 |** Synchronized contractions in cardiac tissue constructs with a cell density of 50 million cells/mL at day 6 (70% iPS-CM, 30% hfCF, GelMA 7.5%).

**Supplementary Video 5 |** Calcium transients of synchronized contractions in cardiac tissue constructs illustrate electromechanical coupling.


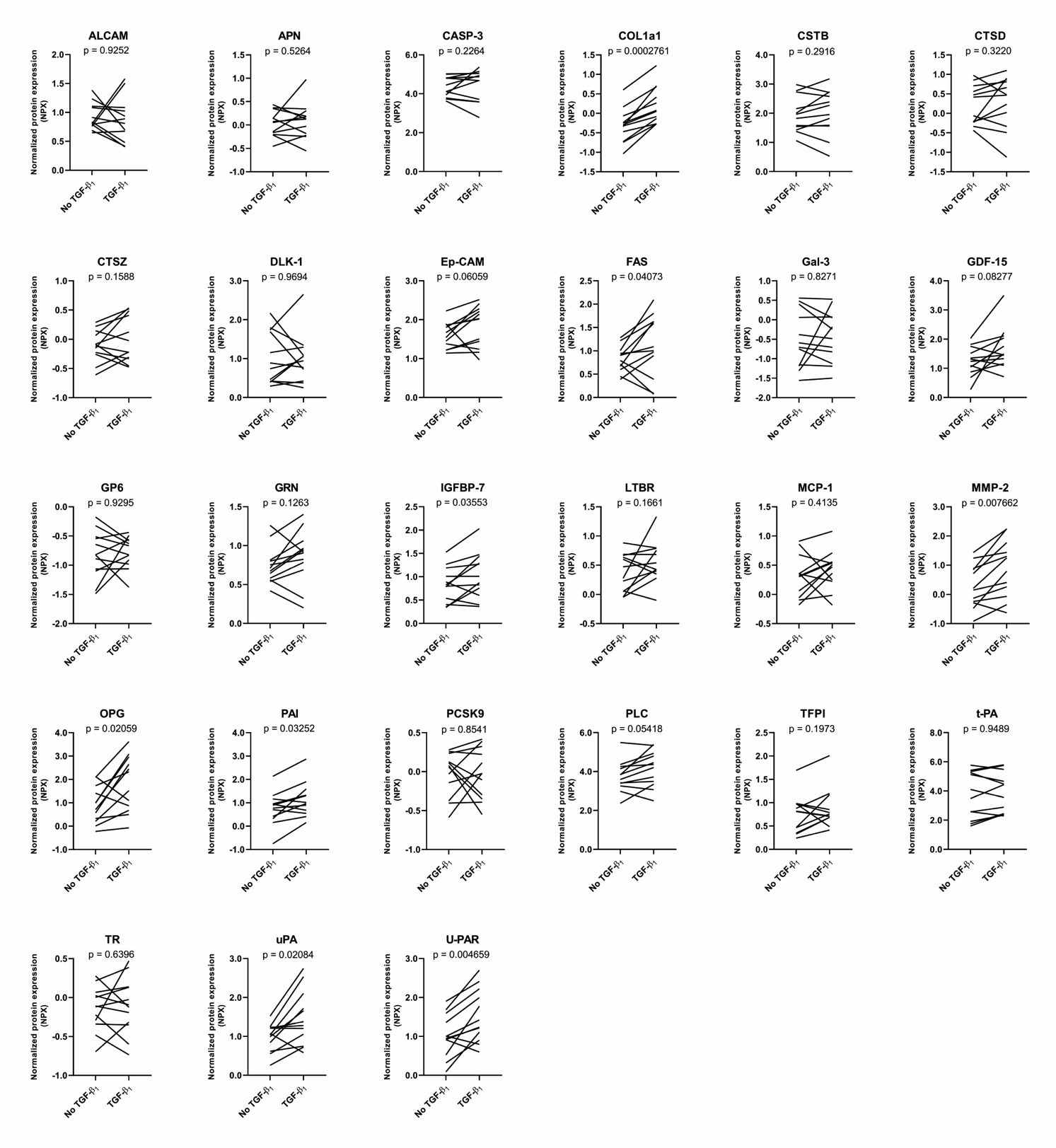


**Supplementary Figure 1 |** **Targeted proteomics of fibrosis in tissue-engineered cardiac constructs.** Normalized protein expression in fibrotic and control conditions for all proteins in Olink Cardiovascular panel 3 that exceeded the level of detection (n = 12). Data are represented as mean normalized protein expression (compared to internal control) per experiment. Statistical analysis was performed using paired two-tailed Student’s t-test.


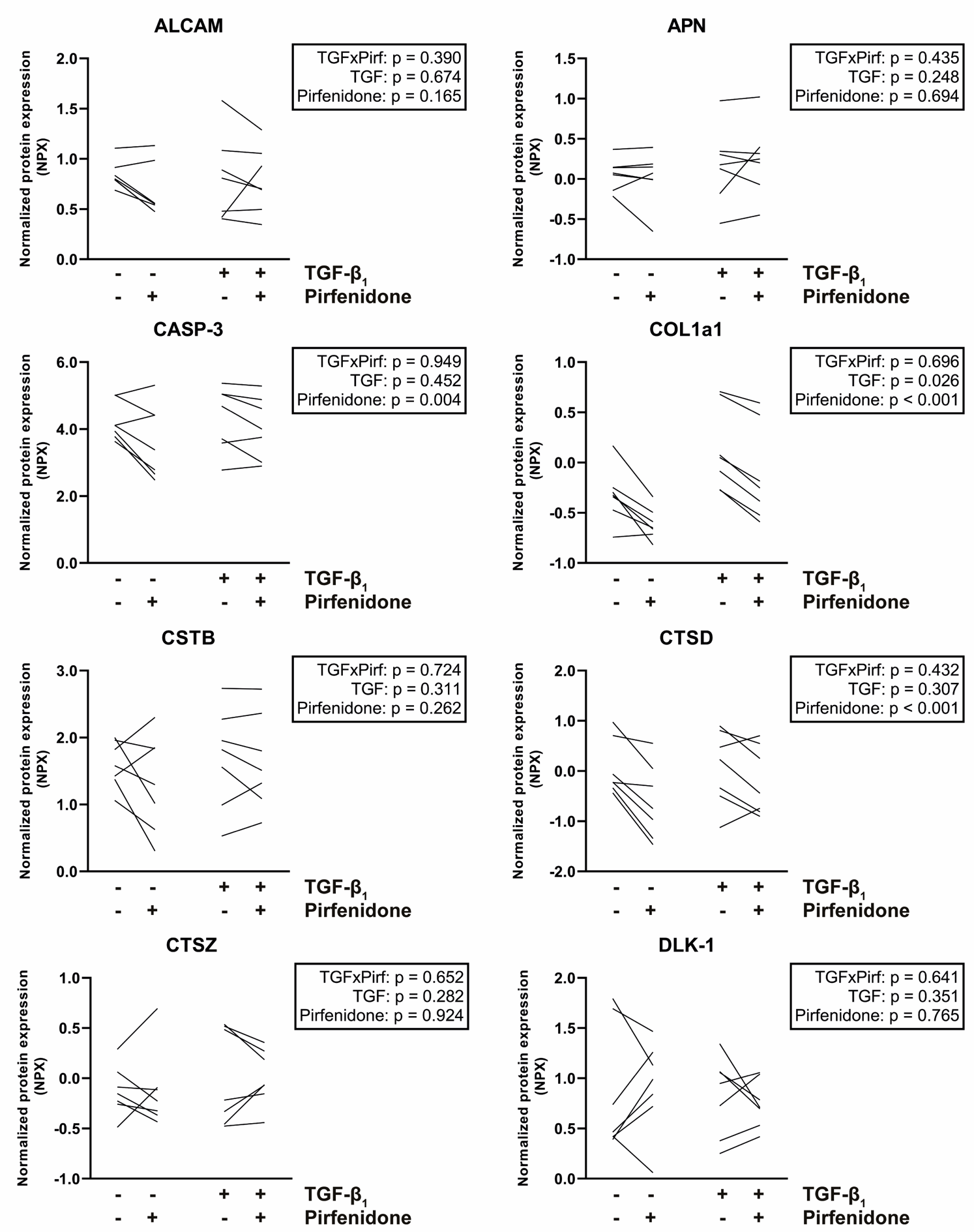

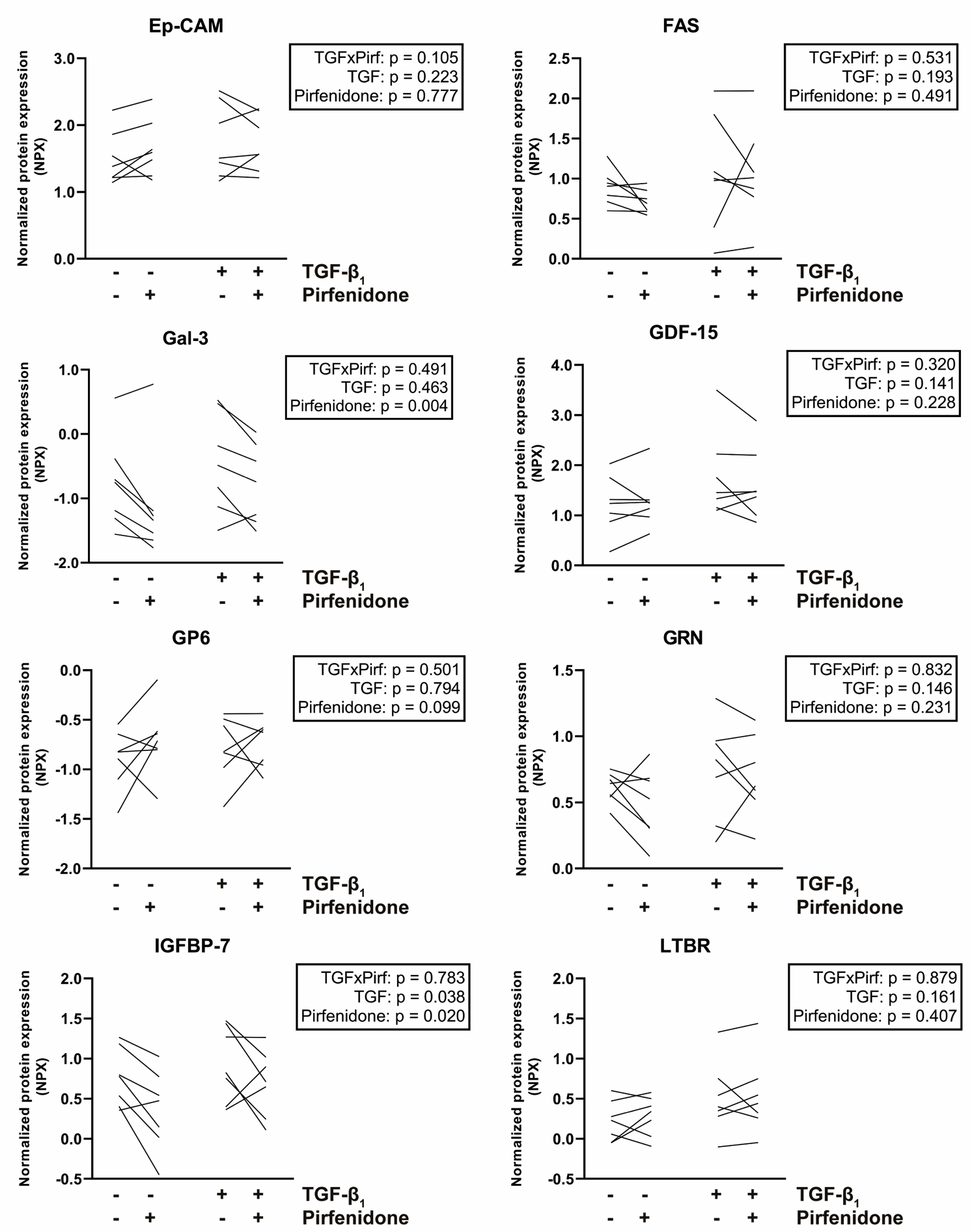

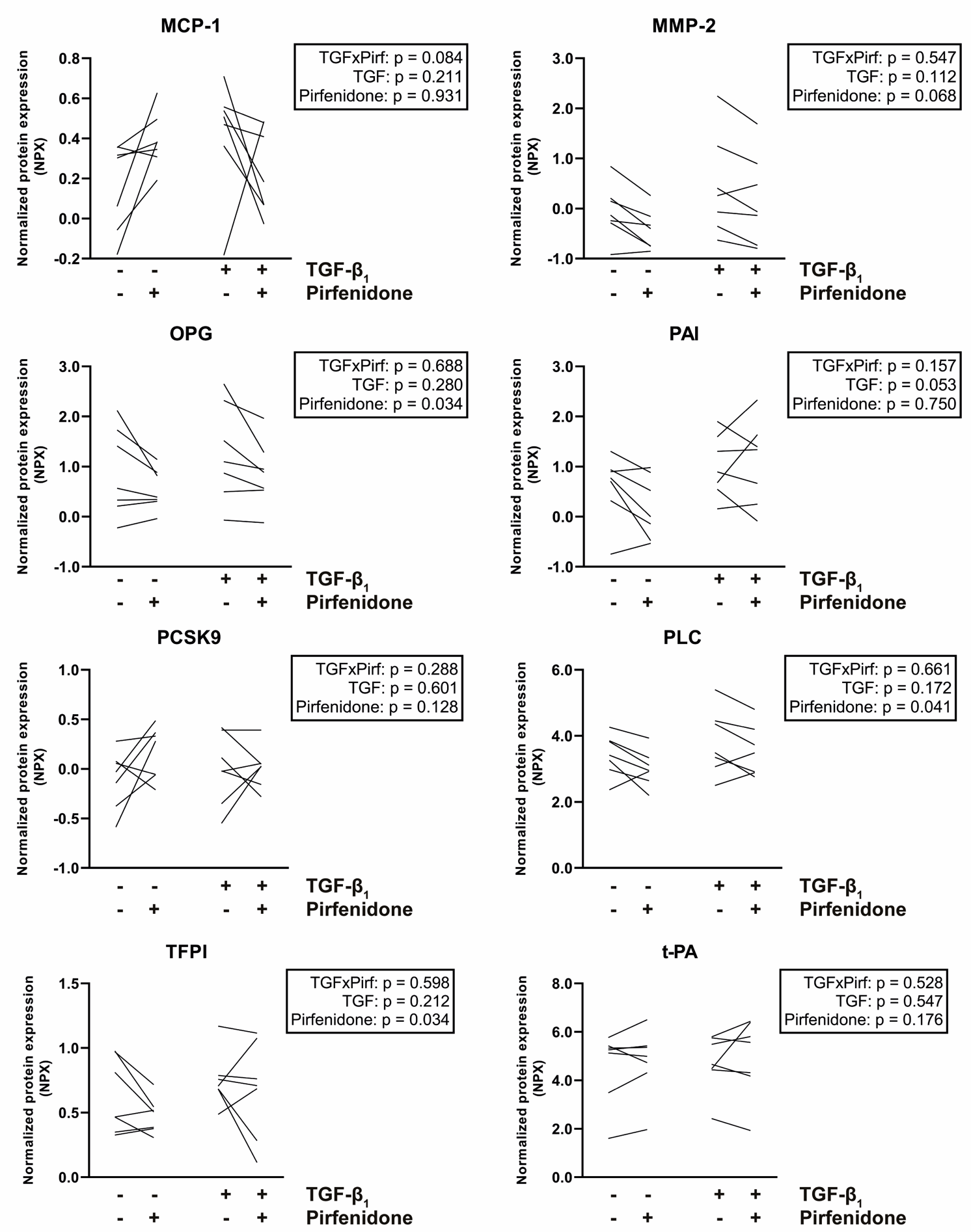

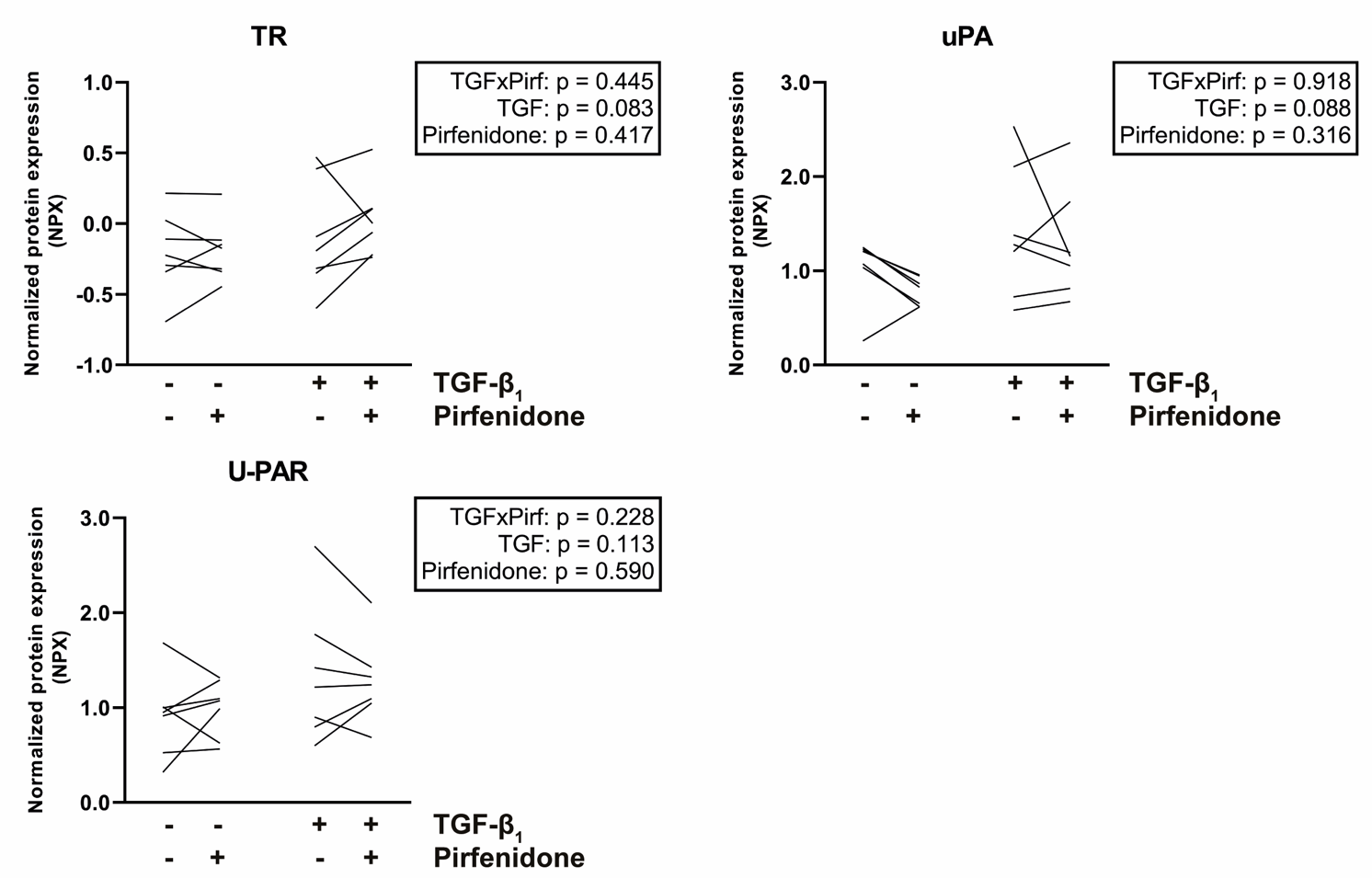


**Supplementary Figure 2 |** **Targeted proteomics of anti-fibrotic treatment in tissue-engineered cardiac constructs.** Normalized protein expression following pirfenidone treatment in fibrotic and control conditions for all proteins in Olink Cardiovascular panel 3 that exceeded the level of detection (n = 7). Data are represented as mean normalized protein expression (compared to internal control) per experiment. Statistical analysis was performed using repeated measures two-way ANOVA. The interaction effect and the main effects are reported in the statistical box on the right side of the graph.

| **Gene** | **Sequence** |
| --- | --- |
| GAPDH | F: 5’-ACAGTCAGCCGCATCTTC-3’ |
|  | R: 5’-GCCCAATACGACCAAATC-3’ |
| α-SMA | F: 5’-AGCCCAGCCAAGCACTG-3’ |
|  | R: 5’-CAAAGCCGGCCTTACAGAG-3’ |
| collagen type 1 alpha chain 1 (COL1a1) | F: 5’-TGCCATCAAAGTCTTCTGC-3’ |
|  | R: 5’-CATACTCGAACYGGAATCCATC-3’ |
| collagen type 3 | F: 5’-AGGGGAGCTGGCTACTTCTC-3’ |
|  | R: 5’-GGACTGACCAAGATGGGAA-3’ |
| periostin | F: 5’-TGCCCTGGTTATATGAGAATGGAAG-3’ |
|  | R: 5’-GATGCCCAGAGTGCCATAAACA-3’ |

**Supplementary Table 1.** Primers used for quantitative real-time polymerase chain reaction (qPCR)

| **Target** | **Manufacturer** | **Cat.#** | **Host** | **Dilution** |
| --- | --- | --- | --- | --- |
| Anti-α-SMA | Sigma Aldrich | A2547 | Mouse | 1:25 |
| Anti-periostin | Abcam | Ab92460 | Rabbit | 1:25 |
| Anti-mouse 488 | Invitrogen | A11029 | Goat | 1:200 |
| Anti-rabbit 568 | Invitrogen | A11036 | Goat | 1:200 |

**Supplementary Table 2.** Antibodies used for immunofluorescence imaging.
